# Inorganic–organic modular silicon and dye-sensitized solar cells and predicted role of artificial intelligence towards efficient and stable solar chargers based on supercapacitors

**DOI:** 10.1038/s41598-024-56302-z

**Published:** 2024-03-13

**Authors:** Ireneusz Plebankiewicz, Krzysztof A. Bogdanowicz, Pawel Kwaśnicki, Wojciech Przybył, Magdalena Skunik-Nuckowska, Pawel J. Kulesza, Agnieszka Iwan

**Affiliations:** 1 Military Institute of Engineer Technology, 136 Obornicka Str, 50-961 Wrocław, Poland; 2 Research and Development Centre for Photovoltaics, ML System S.A., Zaczernie 190G, 36-062 Zaczernie, Poland; 3 Faculty of Chemistry, University of Warsaw, Pasteura 1, 02-093 Warsaw, Poland; 4 Faculty of Natural and Technical Sciences, John Paul II Catholic University of Lublin, ul. Konstantynów 1 H, 20-708 Lublin, Poland

**Keywords:** Silicon solar cell, Dye-sensitized solar cell (DSSC), Solar charger, Modular solar cells, Supercapacitors, Artificial intelligence, Energy science and technology, Engineering

## Abstract

Appropriate and rational management of the energy produced by renewable energy sources is one of the most urgent challenges for the global energy sector. This paper is devoted to the systematic experimental and theoretical studies of a modular solar charger based on silicon and dye-sensitized solar cells as an energy source, and supercapacitor as an energy bank. Using the MathCAD program, I–V characteristics were plotted for both a single cell and a photovoltaic module based on various series-to-parallel connections. To assess the surface quality of the modules, additional tests using a thermal imaging camera were carried out as well. The charging characteristics of the supercapacitor (two series-connected cells with a capacity of 300 F), were determined depending on the parameters of the photovoltaic module as well as considering the influence of the voltage balancing system and control system. The charge, discharge, and recharge characteristics were carefully analyzed to optimize the operating conditions, i.e. the number of photovoltaic cells. To evaluate the stability of parameters with operation time, and their temperature dependence (17–65 °C), solar modules were tested for ten days under Central European weather conditions. Importantly, a comparative analysis of solar chargers based on different configurations of photovoltaic cells showed an increase in electrical parameters for the proposed modular inorganic–organic concept compared to dye-sensitized solar cells produced alone on a rigid substrate. Finally, preliminary assumptions (requirements) were developed regarding the electrical and optical parameters for new dye-sensitized solar cells that could be used in the innovative solar charger instead of silicon cells along with a predicted role of artificial intelligence (AI) in these devices.

## Introduction

The power conversion efficiency (PCE) of amorphous silicon solar cells at a level of 12%, and a relatively large space occupied in relation to the PCE, is considered one of their significant drawbacks^1,2^. On the other hand, dye-sensitized solar cells (DSSC) have a maximum PCE ~ 15% and exhibit low production cost, low consumption of organic materials for their fabrication, and low sensitivity to the angle of solar radiation (they can operate effectively both under the influence of reflected and refracted radiation and in partial shading) compared to silicon technologies. In addition, DSSC are characterized by a smaller amount of CO_2_ produced during the manufacturing process, and a short payback period for the energy used to produce a single DSSC module (Energy Payback Time), which is approx. 0.3 years (*vs*. ca. 2 years for silicon counterpart)^3,4^. Currently, both types of solar cells are produced and are in commercial use. The design of appropriate energy storage facilities is, however, still one of the biggest challenges to enable the competitiveness of PV systems over other energy-related technologies.

In this respect, electrochemical capacitors (supercapacitors) are widely considered auxiliary units for photovoltaic (PV) cells enabling the combination of energy harvesting and energy storage functions within a joint electronic system to store the excess of generated charges, but for most, to stabilize the PV system against fluctuations of solar power. Supercapacitors use the simple physical phenomenon of the formation of an electrical double-layer at the porous electrode/electrolyte interface^5,6^. Since activated carbons are highly microporous materials, the amount of charge that is stored within a certain potential window is incomparably higher than in the case of conventional capacitors whilst the lack of faradaic charge storage mechanisms enables an almost unlimited lifetime (i.e. up to 10^6^ number of cycles or maintenance-free operation up to 10–20 years), ultra-high power density, and reliable performance at low temperatures^5–8^. The energy of supercapacitors is, however, at least one order of magnitude lower in comparison to batteries, and the self-discharge rate is much higher^9^. But, these features are not key limitations in systems coupled to photovoltaic cells by taking into account that (i) as a rule, the supercapacitor is not a primary energy reservoir but only has a supporting function, i.e. providing stabilization of power output, (ii) solar-powered supercapacitor can be recharged in seconds up to few minutes by a solar cell enabling fast compensation of the potential drop due to the self-discharge.

The research focused on the improvement of existing solar cells and supercapacitor technologies is still intensively carried out both in terms of materials engineering, and modifications of the architecture of the devices with a special emphasis placed on the integration of energy harvesting and energy storage components within a single monolithic device^10–18^. However, apart from the technical limitations of the large-scale design, in the case of a failure of one of the modules, the entire panel would probably need replacement. Therefore, coupling two separate units (PV and supercapacitor) conventionally, i.e. employing a wire connection and appropriately designed control system still seems to be a rational option. However, there is still relatively little work on the design and characteristics of devices such as solar chargers which are tested under natural (environmental) conditions. For example, Plebankiewicz et al.^19^ and Skunik-Nuckowska et al.^20^ presented solar chargers based on commercial components, i.e. silicon cells and double-layer supercapacitors, as well as commercial silicon cells coupled to novel hybrid supercapacitor banks. It was possible to achieve efficiency of energy recovery from the supercapacitors at the level of 0.93, which was a very good result for modular construction^19^. The practical significance of hybrid redox electrolyte-based supercapacitors as the main component of a solar charger was presented in^20^. Introducing a redox electrolyte (an aqueous potassium iodide) instead of a conventional non-electroactive salt solution allowed for a double increase in the capacity and energy of a capacitor. The system charged successfully in approx. 2 h in the temperature ranging from 15 to 25 °C, hence similar to the climate present at European latitudes for 2nd and 3rd quarter of the year. Plebankiewicz et al.^21^ developed *a know-how* package for the solar charger design based on novel dye-sensitized solar cells and a 60 F/3 V supercapacitor bank constructed from appropriately series-to-parallel-connected CR2032 coin cells. Using the engineering concepts for the construction of microprocessor measuring and control systems and Artificial Intelligence algorithms to control the environmental conditions, fully functional solar chargers capable of reliable operating at a temperature of 15–25 °C were obtained. On the whole, the chargers reported in^20^, and^21^ were capable of maintaining the power supply for approx. 2 min (in the case of a silicon PV module), and 22 s (for the DSSC module) which can be considered a satisfactory result for some of the emergency power supply applications such as in the SOS transmitters or igniters.

Moreover, some key solutions have been patented and are currently in the commercialization phase^22–24^. The intellectual property consists of the details regarding (i) the optimization of electrical connections between the solar module and supercapacitor unit, (ii) the energy conversion pathways reducing the energy loss during the adjustments between the input and output current characteristics, and (iii) the protection solutions preventing against overcurrent damage during the energy peak generation form the photovoltaic module. The product with a weight of less than 1 kg, embodied in durable and resistant to damage enclosure and equipped with USB port, is currently being produced by ML System and is commercially available^25^.

Although, DSSC are beneficial over silicon technologies in several crucial manners, there is still more research required regarding the possible recycling and improving the end-of-life^26,27^. There have been also some works suggesting modification of design to fit into the technological gap of solar cells and combine them as one device with energy storage units such as supercapacitors. There have been proposed several architectures of so-called photo-supercapacitors which include two or three-electrode structures, coaxial connections, or twisted and parallel fibrous structures. However, it was noticed that even though the progress made in the last two decades is quite significant, coupled DSSC-supercapacitor devices are still in the development stage^12,13,28–30^.

Finally, the latest trends in the field of renewable energy sources, including the deployment of artificial intelligence (AI) should be mentioned as well. Optimizing the distribution and storage of electricity using AI algorithms is of great importance for rational energy management, for instance, the predictions of supply and demand. Farhadi et al. focused on a subfield of AI, i.e. machine learning (ML)^31^. ML is based on specific algorithms that analyze the data using: regression, classification, clustering dimensionality reduction, or provability. However, the use of ML in material science requires definite refinement due to sufficient labeling, the available amount of unified data, and the well-studied relation between materials structure and expected properties. Maleki et al. raised similar concerns regarding the accuracy of AI approach^32^. They compared machine learning, deep learning, and artificial neural networks for the prediction of materials’ properties for clean energy, and have shown how difficult is to offer a universal method for a vast group of materials with different applicability.

On the other hand, Adamu et al.^33^ proposed AI-navigated development of high-performance energy storage materials, including those used in supercapacitors. The input parameters such as the pore surface area of electrode material, and physicochemical properties of electrolytes were used to predict the electrical parameters of the training models with high accuracy. It shows the great importance of AI in understanding the electrochemical behavior of charge storage devices and saving time consumed for the preparation of materials and gathering the research data. The development of new AI models along with providing free access to materials databases are, however, the greatest challenges facing AI.

The main goal of our work was the construction of solar chargers based on modular silicon cells and DSSCs coupled to a supercapacitor bank. In contrast to hybrid solar cells, i.e. based on a heterojunction formed between organic and inorganic semiconductive materials^34–37^, here the individual elements can be replaced like the effect of Lego blocks or a puzzle. Following this direction, this work presents in detail the characteristics of both the individual components of the charger and the final system to create a library of devices generating and storing electricity in a modular form. In details we investigated:

- The influence of electrical parameters such as series resistance, current, stability on the I–V characteristics over time and temperature,
- The charging characteristics of the supercapacitor based on the analysis of the schematic diagram of the photovoltaic module in a series–parallel configuration,
- The influence of the voltage balancing system and the control system on the charging characteristics of supercapacitors with constant current up to the rated voltage,
- The optimal number of photovoltaic cells in a series–parallel configuration of solar charger,
- The supercapacitor storage in terms of charging, discharging, and recharging in a symulated work cycle with one of many possible loads (receiver in the form of an SOS transmitter).

The article presents the results of both theoretical and experimental studies on the components of a solar charger along with the ecological, economic, and aesthetic aspects of the proposed model which are taken into account as well. Finally, finding a path for future works on the construction of efficient solar chargers based solely on DSSC and supercapacitors was intended as well.

## Materials and methods

The study involved commercial DSSC with serial numbers SN 080120SN01 and SN 080120SN03 (Solarix, Aubonne, Switzerland), two AM-5706 amorphous silicon cells on a glass substrate (PANASONIC), and four AT-7565 amorphous silicon cells on a thin foil substrate (PANASONIC) together with four supercapacitors (type XB, EATON) with capacity C = 400 F and operating voltage V_C_ = 2.5 V.

The measurement of the current–voltage characteristics (I–V) of photovoltaic cells was performed on SS150AAA solar radiation simulator coupled with I–V measurement system (Tracer SS I–V CT-02 integrated with a Keithley SM2401 sourcemeter). The temperature was ranging from 17 to 65 °C.

Thermal behavior was observed upon applying a potential and using a thermographic camera (VIGOcam v50, VIGO System S.A, Ozarow Mazowiecki, Poland). A multichannel potentiostat/galvanostat (PGStat Autolab M101, Metrohm, Barendrecht, Nederland) was used in the experiments.

### Construction of a solar charger based on silicon solar cells and dye-sensitized solar cells coupled to supercapacitor

**Figure 1:**
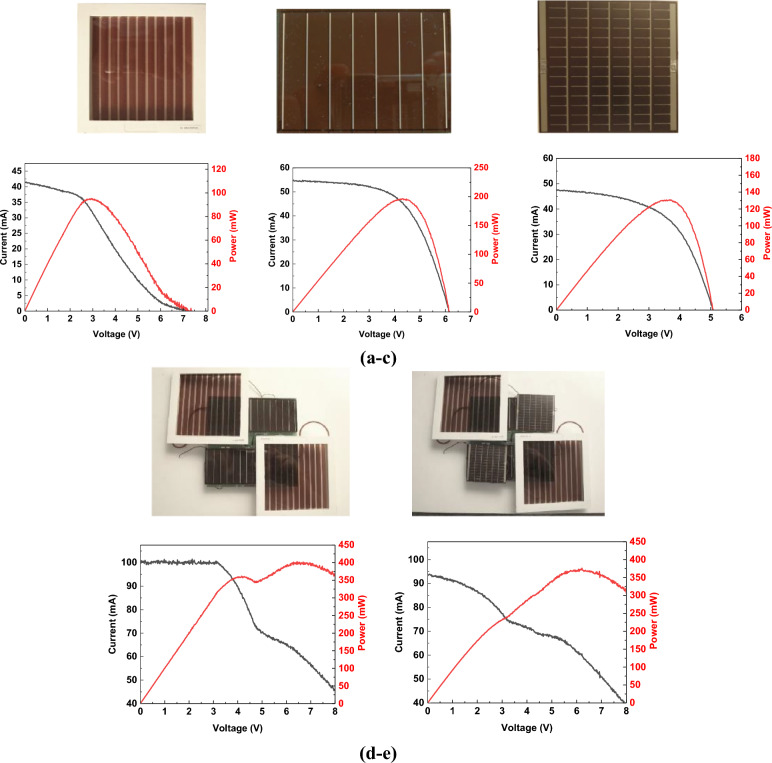
Photographs and I–V characteristics of investigated solar cells: (**a**) DSSC with photosensitive field dimensions of 91 mm × 91 mm, (**b**) an amorphous silicon cell on a glass substrate with photosensitive field dimensions of 68 mm × 48 mm, (**c**) an amorphous silicon cell on a flexible substrate with photosensitive field dimensions of 56 mm × 55 mm, (**d**) two DSSC and two silicon cells on a glass substrate with a total size of the photosensitive field of 224.6 cm^2^ and (**e**) two DSSC and two silicon cells on a plastic substrate with a total size of the photosensitive field of 244.7 cm^2^.

The functional model was made based on the schematic diagrams (discussed below) of systems composed of a regulated current source, voltage balancing system, and supercapacitor charging voltage control system. A schematic diagram of the supercapacitor charging system with a constant current I_z_ = 0.3 A was made in the unified environment for designing electronic systems Altium Designer by computer controls. The photovoltaic measurements were performed for the following solar cells:

(i) DSSC with photosensitive field dimensions of 91 mm × 91 mm (Fig. 1a),
(ii) An amorphous silicon cell on a glass substrate with photosensitive field dimensions of 68 mm × 48 mm (Fig. 1b),
(iii) An amorphous silicon cell on a flexible substrate (thin film) with photosensitive field dimensions of 56 mm × 55 mm (Fig. 1c)

and for solar modules in a series–parallel connection:

(i) Two DSSC and two silicon cells on a glass substrate with a total surface area of the photosensitive field of 224.6 cm^2^ (Fig. 1d),
(ii) Two DSSC and two silicon cells on a flexible substrate with a total surface area of the photosensitive field of 244.7 cm^2^ (Fig. 1e).

The solar cells and corresponding modules were tested, and then the average parameter values were determined and the average I–V characteristics were plotted for individual cell types (see Fig. 1). Table 1 presents a summary of the average PV parameters of the tested cells (modules) at the average light intensity E = 970 W/m^2^, during the measurements performed at ambient temperature T = 25 °C.

**Table 1:** Summary of the average PV parameters of the tested cells (modules) at E = 970 W/m^2^, T = 25 °C.

Type of solar cell	DSSC	AM-5706	AT-7565	DSSC/AM-5706	DSSC/AT-7565
Total photosensitive field	91 × 91 mm	68 × 48 mm	56 × 55 mm	224.6 cm^2^	224.7 cm^2^
PARAMETERS:					
short-circuit current I_sc_ [mA]	41.5	54.6	48.4	99.7	93.5
open circuit voltage V_oc_ [V]	0,0072	6.1	5.0	8.0	8.0
maximum current I_max_ [mA]	32.6	45.6	37.3	61.9	60.1
maximum voltage V_max_ [V]	2.9	4.3	3.4	6.4	6.1
maximum power P_max_ [mW]	94.8	195.8	126.9	398.8	372.0
Fill Factor FF [–]	0.3	0.6	0.53	0.5	0.5
Power Conversion Efficiency PCE [%]	1.2	5.7	3.7	1.8	1.7
series resistance R_s_ [Ω]	466.4	25.6	26.6	122.1	55.5
shunt (parallel) resistance R_sh_ [kΩ]	2.5	5.6	644.0	0.1041	240.2

The current–voltage characteristics were averaged over three independent measurements whilst the stability of electrical parameters over time and their dependence on temperature (17–65 °C), were tested for ten days.

**Figure 2:**
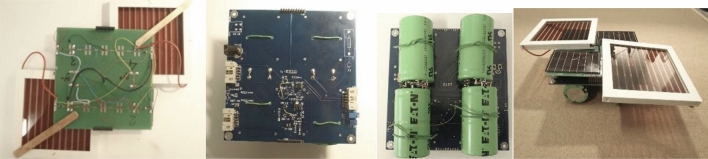
Photographs of constructed modular solar cells based on DSSC and silicon solar cells (AM-5706) on glass substrate, current source block, voltage control system, supercapacitors block (capacity C = 400 F, operating voltage V_C_ = 2.5 V), and final solar charger (side view).

The next step in the construction of the solar charger was to analyze the operational properties of the components. Before assembling all elements, were checked for compliance and quality with the provided documentation by inspecting the characteristic dimensions of the circuit, confirming the absence of contamination on the surface, i.e. the residue of mechanical processing, dust, and laminate shreds. After confirming that the tiles were free from defects and their quality was satisfactory, the assembly of the elements began (Fig. 2).

### Application of the MathCAD program to visualization of the physical model in graphical form

The next step was the visualization of the physical model in graphical form using computer-aided design including: (i) modeling the solar characteristics of a single photovoltaic cell, and a PV module composed of single photovoltaic cells operating in a series–parallel connection, and (ii) analysis of the influence of individual parameters, i.e. series resistance R_s_, current I_ph_ and temperature T on the solar characteristics of the PV module. Before visualizing, the solar characteristics of a single photovoltaic cell were simulated using a mathematical model. For this purpose, the MathCAD program was used to perform quick mathematical calculations and enable the preparation of legible documentation at the same time.

#### Mathematical equations used in the simulation study

The mathematical simulations were based on the following equations^38–45^:

The equation describing the current dependencies in PV:

1 $$I={I}_{ph}-{I}_{d}-{I}_{rsh}$$

The equation of diode current:

2 $$I={I}_{o}\left[{e}^{\left(\frac{q{V}_{d}}{akT}\right)}-1\right]$$

The equation of resistance current as resistance and potential dependence:

3 $${I}_{sh}=\frac{{V}_{sh}}{{R}_{sh}}$$

The equation of diode’s potential:

4 $${V}_{d}={V}_{sh}=V+I{R}_{s}$$

where: I—current of the PV panel, I_ph_—current generated by the photovoltaic effect, I_o_—diode saturation current, I_d_—diode current, V_sh_—potential of the shunt resistance of the PV cell, I_sh_—current of the shunt resistance of the PV cell, V_d_—diode potential.

Constants used in modeling:

Boltzmann constant: k = 1.38 × 10^–23^ [J/K]

elemental charge: q = 1.602 × 10^–19^ [C]

potential barrier: v_q_ = 1.12 q[V/C]

the temperature at Standard Temperature Conditions: T_0_ = 298.15 [K]

the maximum solar radiation power density: G_0_ = 1000 [W/m^2^]

the maximum of light intensity: E_0_ = 126,582.29 lx

#### Predefined data for single PV cell

Open circuit potential: V_OC_≡540 mV

Short circuit current: I_SC_≡ 500 mA

Series resistance: R_s_≡ 0.01 Ω

Shunt resistance: R_sh_≡ 1 kΩ

Diode quality factor: n ≡ 1.5

Temperature factor: α ≡ 0.0002 A/K

Number of cells: PVn: = 1

Maximum peak voltage (single cell): V_mpv_ = PVn V_oc_ = 0.54 V

Voltage range (single cell): V_pv_ = 0 V, 0.01V_oc_…PVn 1.1 V_oc_

Number of cells connected in series: OPV_nS ≡ 20

Number of parallel connections: OPV_nR ≡ 3

Maximum peak voltage (panel): V_mpv_ = OPV_nS V_oc_ = 10.8 V

Voltage range (panel): V_pv_ = 0 V, 0.01V_oc_…OPV_nS 1.1 V_oc_

#### Model equations

5 $${E}_{v}\left(T,V\right)={e}^{\left(\frac{q}{nkT{PV}_{n}}\right)V}$$

6 $${E}_{v}\left(T,V\right)={e}^{\left(\frac{q}{nkTO{PV}_{nS}}\right)V}$$

7 $${I}_{ph}\left(T,G\right)=\frac{G}{{G}_{0}}\left[{I}_{sc}+\propto \left(T-{T}_{0}\right)\right]$$

8 $${I}_{ph}\left(T,E\right)=\frac{E}{{E}_{0}}\left[{I}_{sc}+\propto \left(T-{T}_{0}\right)\right]$$

9 $${I}_{D}\left(T,I,{R}_{s},V\right)=\frac{{I}_{sc}}{{E}_{v}\left(T,{U}_{mpv}\right)-1}{\left(\frac{T}{{T}_{0}}\right)}^{3}{E}_{v}\left(T,{v}_{q}\right)\left({E}_{v}\left(T,V+I{R}_{s}\right)-1\right)$$

10 $$I\left({R}_{s},T,G,V\right)=root\left({I}_{ph}\left(T,G\right)-{I}_{D}\left(T,{I}_{sc},{R}_{s},V\right)-\frac{{V+I}_{sc}{R}_{s}}{{R}_{sh}}-{I}_{sc},{I}_{sc}\right)$$

11 $$I\left({R}_{s},T,E,V\right)=root\left({OPV}_{nR}\left(T,E\right)-{OPV}_{nR}{I}_{D}\left(T,{I}_{sc},{R}_{s},V\right)-\frac{{V+I}_{sc}{R}_{s}}{{{OPV}_{nR}R}_{sh}}-{I}_{sc},{I}_{sc}\right)$$

root equation I(R_s_, T, G, V) and I(R_s_, T, E, V) for V_mpv_:

root(I(R_s_, T_0_, G_0_, V_mpv_)V_mpv_) = 0.54 V

root(I(R_s_, T_0_, E_0_, V_mpv_)V_mpv_) = 10.797 V

The range of changes in solar radiation power (G_0_), in illumination intensity (E_0_) and series resistance range (R_s_) along with the temperature range (T_0_) for the solar cell are presented below, respectively.

$$ \left( {\begin{array}{*{20}c} {{\text{G}}_{1} } \\ {{\text{G}}_{2} } \\ {{\text{G}}_{3} } \\ {{\text{G}}_{4} } \\ {{\text{G}}_{5} } \\ {{\text{G}}_{6} } \\ {{\text{G}}_{7} } \\ {{\text{G}}_{8} } \\ {{\text{G}}_{9} } \\ {{\text{G}}_{10} } \\ \end{array} } \right): = \left( {\begin{array}{*{20}c} {0.1} \\ {0.2} \\ {0.3} \\ {0.4} \\ {0.5} \\ {0.6} \\ {0.7} \\ {0.8} \\ {0.9} \\ 1 \\ \end{array} } \right).{\text{G}}_{0} \left( {\begin{array}{*{20}c} {{\text{E}}_{1} } \\ {{\text{E}}_{2} } \\ {{\text{E}}_{3} } \\ {{\text{E}}_{4} } \\ {{\text{E}}_{5} } \\ {{\text{E}}_{6} } \\ {{\text{E}}_{7} } \\ {{\text{E}}_{8} } \\ {{\text{E}}_{9} } \\ {{\text{E}}_{10} } \\ \end{array} } \right): = \left( {\begin{array}{*{20}c} {0.1} \\ {0.2} \\ {0.3} \\ {0.4} \\ {0.5} \\ {0.6} \\ {0.7} \\ {0.8} \\ {0.9} \\ 1 \\ \end{array} } \right).{\text{E}}_{0} \quad \left( {\begin{array}{*{20}c} {{\text{R}}_{{{\text{s}}1}} } \\ {{\text{R}}_{{{\text{s}}2}} } \\ {{\text{R}}_{{{\text{s}}3}} } \\ {{\text{R}}_{{{\text{s}}4}} } \\ {{\text{R}}_{{{\text{s}}5}} } \\ {{\text{R}}_{{{\text{s}}6}} } \\ {{\text{R}}_{{{\text{s}}7}} } \\ {{\text{R}}_{{{\text{s}}8}} } \\ {{\text{R}}_{{{\text{s}}9}} } \\ {{\text{R}}_{{{\text{s}}10}} } \\ \end{array} } \right): = \left( {\begin{array}{*{20}c} {0.01\Omega } \\ {0.02\Omega } \\ {0.03\Omega } \\ {0.04\Omega } \\ {0.05\Omega } \\ {0.06\Omega } \\ {0.07\Omega } \\ {0.08\Omega } \\ {0.09\Omega } \\ {0.1\Omega } \\ \end{array} } \right)\quad \left( {\begin{array}{*{20}c} {{\text{T}}_{1} } \\ {{\text{T}}_{2} } \\ {{\text{T}}_{3} } \\ {{\text{T}}_{4} } \\ {{\text{T}}_{5} } \\ {{\text{T}}_{6} } \\ {{\text{T}}_{7} } \\ {{\text{T}}_{8} } \\ {{\text{T}}_{9} } \\ {{\text{T}}_{10} } \\ \end{array} } \right): = \left( {\begin{array}{*{20}c} {0.93292} \\ {{0}{\text{.94969}}} \\ {{0}{\text{.96646}}} \\ {{0}{\text{.98323}}} \\ 1 \\ {1.01677} \\ {{1}{\text{.03354}}} \\ {{1}{\text{.05031}}} \\ {{1}{\text{.06708}}} \\ {{1}{\text{.08385}}} \\ \end{array} } \right).{\text{T}}_{0} $$

## Results and discussions

### Optimal combination of supercapacitor and solar cell

The implementation of the article's objective began with the development of initial assumptions as to the parameters of individual components of the model based on the parameters of selected components such as a photovoltaic cell, a supercapacitor, a supercapacitor charging and discharging module, and a control and measurement system. The critical parameters for the photovoltaic cell-supercapacitor system are (i) the charging current of the supercapacitor determining the output current from the photovoltaic cell; (ii) the operating voltage of supercapacitors V_c_, which ranges from 2.5 to 2.8 V or from 5.0 to 5.6 V in the case of 2 supercapacitors connected in series; (iii) supercapacitor discharge time and current through I_o_ load, which determines the supercapacitor's capacity, and thus affects the supercapacitor charging time and current; (iv) capacity of the supercapacitor (the higher the capacity, the longer the charging time).

Possible energy to be stored in such a system can be written as:

12 $$E= \frac{{V}_{O}^{2}{C}_{w}}{2}$$

where: V_o_—maximum charging voltage; C_w_—resultant capacity of the supercapacitor battery; n—number of supercapacitors.

For a supercapacitor battery system connected in series (Eq. 13) and parallel (Eq. 14), respectively, the capacity C_W_ can be described as:

13 $${C}_{w}=\frac{C}{n}$$

14 $${C}_{w}=Cn$$

Table 2 shows the dependence of energy on voltage and capacity of supercapacitors in a series and parallel arrangement.

**Table 2:** The dependence of energy on voltage and capacity of supercapacitors in a series and parallel arrangement.

No. of supercapacitors, n	Series connection			Parallel connection		
	Working voltage V_o_ [V]	Resultant capacity C_w_ = C/n [C]	Energy E [Ws]	Working voltage V_o_ [V]	Resultant capacity C_w_ = C × n [C]	Energy E [Ws]
1	2.5	300	937.5	2.5	300	937.5
2	5.0	150	1875.0	2.5	600	1875
3	7.5	100	2812.5	2.5	900	2812.5
4	10.0	75	3750.0	2.5	1200	3750.0
5	12.5	60	4687.5	2.5	1500	4687.5
6	15.0	50	5625.0	2.5	1800	5625.0

**Figure 3:**
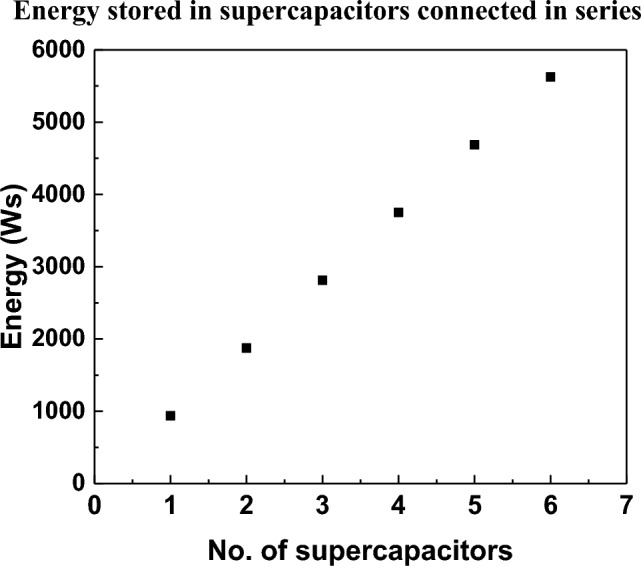
Graph of energy dependence on the number of supercapacitors with rated capacity C = 300 F and operating voltage V_o_ = 2.5 V.

**Figure 4:**
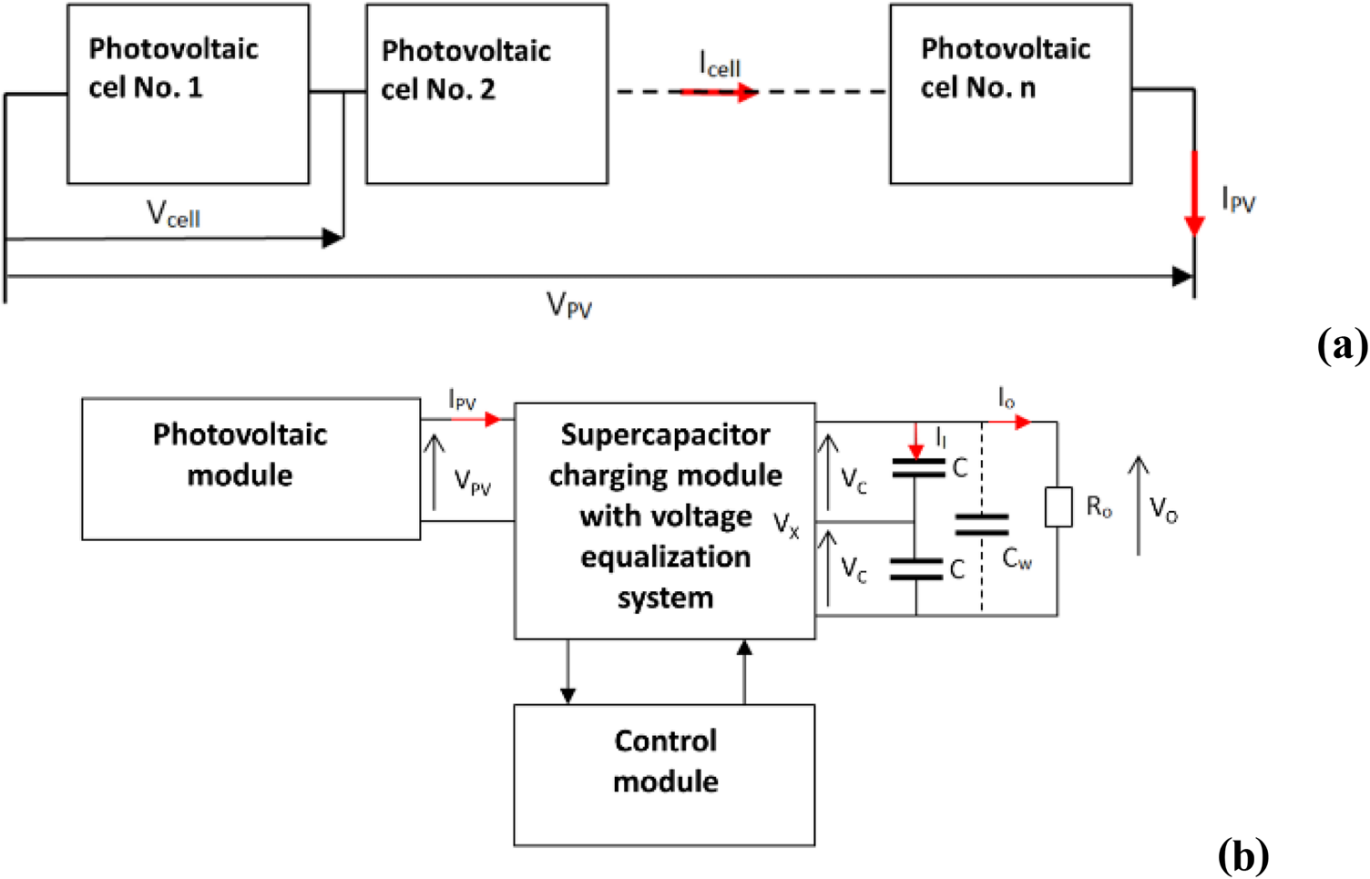
(**a**) A battery of photovoltaic cells connected in series, I_PV_ = I_cell_, V_PV_ = n × V_cell,_ (**b**) Block diagram of a photovoltaic module and 2 supercapacitors connected in series, where C_w_: resultant capacitance of the supercapacitor C_w_ = C/n; n: number of supercapacitors in the battery.

Figure 3 shows that the number of supercapacitors in the battery system linearly affects the amount of energy stored. The only difference is that in a series connection the operating voltage of the entire system increases, and in a parallel connection the current increases. This is important from the point of view of the receiver, which is characterized by a specific voltage (V_po_) and operating current (I_po_). The results shown further correspond to the system of a photovoltaic cell with a bank of supercapacitors connected in series. Since the output voltage of a single photovoltaic cell is about V_cell_ = 0.56 V, to obtain at least the UPV voltage of 5 ÷ 7 V at the input of the supercapacitor battery charging system, a series-connected photovoltaic battery system must be used (Fig. 4a). The schematic diagram of the system of photovoltaic cells connected in series to charge the battery of supercapacitors is presented (Fig. 4b).

**Figure 5:**
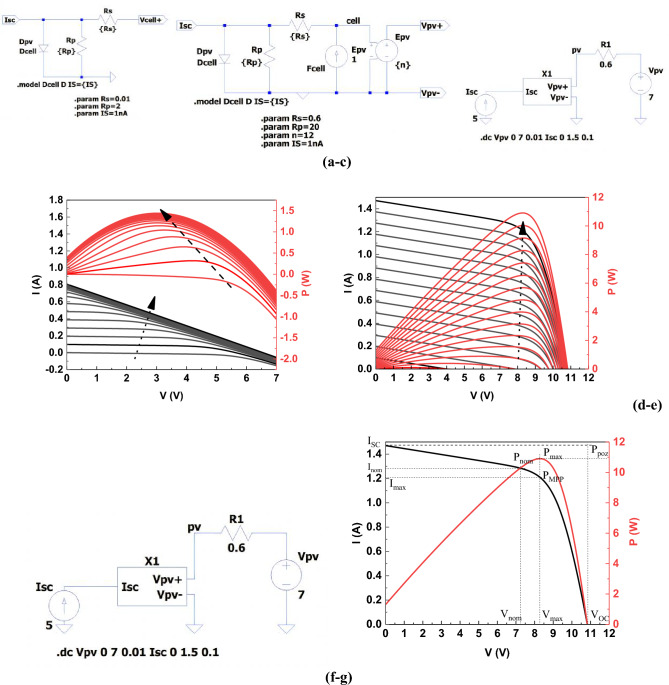
(**a**) Equivalent diagram of a photovoltaic cell (single diode model), where: R_s_: series resistance of the photovoltaic cell; R_p_: parallel (shunt) resistance of the photovoltaic cell; I_d_: diode current; I_sc_: photocurrent proportional to solar radiation intensity; V_cell+_: output voltage of the photovoltaic cell. (**b**) A placeholder diagram of the module symbol of 12 photovoltaic cells connected in series. (**c**) An equivalent diagram used to simulate a photovoltaic module composed of 12 photovoltaic cells connected in series, where: X1: symbol of 12 photovoltaic cells connected in series, I_sc_: is a current source whose current, as a result of the photovoltaic phenomenon, is directly proportional to the intensity of solar radiation. (**d**) Characteristics I = f(V) and P = f(V) of a photovoltaic module composed of 12 and 20 PV cells (**e**) photovoltaic cells connected in series. (**f**) An equivalent diagram of a photovoltaic module consisting of 20 photovoltaic cells connected in series, where X1: the symbol of 20 photovoltaic cells connected in series. (**g**) Characteristics I = f(V) and P = f(V) of a photovoltaic panel composed of a battery of 20 photovoltaic cells connected in series.

The phenomena occurring in a photovoltaic cell can be described using a single-diode or two-diode model. The simplest and most commonly used model is the one-diode model (Fig. 5a). The two-diode model is used less often due to the rather complicated numerical calculations.

The electrical diagram (equivalent) of the single diode model contains discrete elements whose behavior is precisely known. Such a model can be simulated with any program for the simulation of electrical circuits. In this study, the Analog Device's LTSpice, which is available free of charge and has a wide range of electronic component models was used for simulation procedures. The program also allows to define own models that can be used as symbols in more complex electrical diagrams. An example of such a symbol representing 12 photovoltaic cells connected in series is shown in Fig. 5b and c. Using the above model, a simulation was carried out, and as a result, the following characteristics were obtained (Fig. 5d). The above graphs show that with a photovoltaic module consisting of 12 photovoltaic cells connected in series, the UPV voltage > 7 V cannot be received to charge the battery of 2 supercapacitors connected in series. Therefore, a photovoltaic module composed of 20 photovoltaic cells connected in series was simulated (Fig. 5f) and the following characteristics were obtained (Fig. 5e).

To sum up, the above presents block diagrams of photovoltaic cell—supercapacitor modules in systems where a single supercapacitor and a supercapacitor bank are charged. The energy dependence on the voltage and capacity of supercapacitors is also presented, as a battery in a series and parallel system. It has been shown that the number of supercapacitors in the battery system linearly affects the amount of energy stored, and in series connection, the operating voltage of the entire system increases. Therefore, a photovoltaic cell system with a battery of supercapacitors connected in series was selected for further consideration. A single-diode model of a photovoltaic cell was presented and the above cell was simulated in an electrical circuit simulation program. The obtained graphs (Fig. 5d,e) showed that with a photovoltaic panel consisting of a battery of 12 photovoltaic cells connected in series, V_PV_ voltage > 7 V is not reachable to enable charging of a battery of 2 supercapacitors connected in series. Therefore, a second simulation of a photovoltaic panel consisting of a battery of 20 photovoltaic cells was performed and the I–V and P–V characteristics were obtained (Fig. 5g). Then, the following values characterizing a given photovoltaic system were determined: short-circuit current I_SC_ at V_oc_ = 0; electromotive force V_OC_ at I_sc_ = 0; nominal power point P_nom_; apparent P_poz_ power point; maximum power point P_max_; Fill Factor (FF) and power conversion efficiency (PCE). It enabled to calculate the following parameters: P_nom_ = $$\begin{gathered} {7}.{33}\;{\text{V }} \times { 1}.{29}\;{\text{A }} = { 9}.{46 }\;{\text{W}};{\text{ P}}_{{{\text{poz}}}} = { 1}0.{83}\;{\text{V }} \times { 1}.{47}\;{\text{A }} = { 15}.{92}\;{\text{W}};{\text{P}}_{{{\text{max}}}} = { 8}.{5}\;{\text{V }} \times { 1}.{47}\;{\text{A }} = { 12}.{49}\;{\text{W}}; \hfill \\ {\text{FF }} = { 9}.{46 }/{ 15}.{92}^{{}} = \, 0.{59}\;{\text{and}}\;{\text{PCE }} = \, \left( {{9}.{46}/\left( {{1}000*0.0{15}} \right)} \right) \, \times { 1}00\% \, = { 63 }\% . \hfill \\ \end{gathered}$$

**Figure 6:**
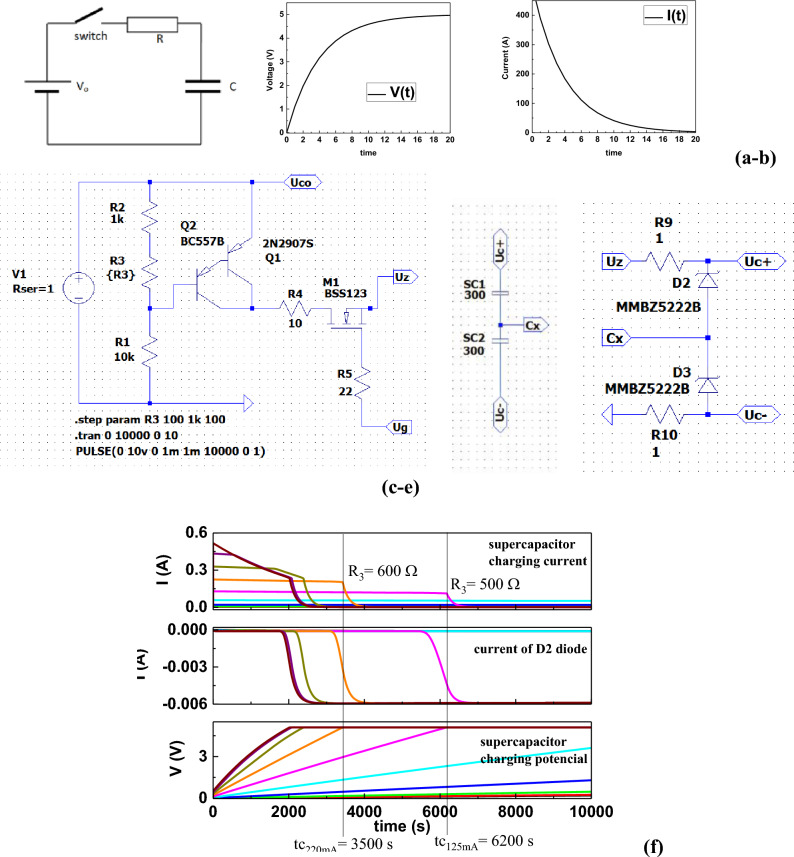
Capacitor charging system with alternating current (**a**) and capacitor charging current characteristics (**b**), Adjustable current source (**c**), and a battery of series-connected supercapacitors (**d**) with the voltage balancing system (**e**). Characteristics of the supercapacitor voltage balancing system (**f**), where: a) supercapacitor charging current I_sc_; b) current I_DZ_ of diode D2; c) supercapacitor charging voltage V_sc_; t_c_: time of charging the supercapacitor to an operating voltage of 2.4 V.

As already mentioned, the number of supercapacitors in the battery system linearly affects the amount of energy stored. In a series connection, the operating voltage of the entire system increases. Since the receiver is characterized by a specific voltage (V_o_) and operating current (I_o_), this is important when designing the entire photovoltaic battery-supercapacitor module. The simplest capacitor charging system is built from a voltage source V_o_ connected to the capacitor C through a resistor R (Fig. 6a).

When the capacitor is charged, the potential on its electrodes changes according to the formula:

15 $${\text{V}}\left(t\right)={V}_{0}(1-{e}^{\frac{-t}{\tau }})$$

where: τ: capacitor charging constant τ = R × C; R: internal resistance of the capacitor R = 12 mΩ; C: capacity of the capacitor bank C = 150 F.

This system has a basic drawback—the charging current I(t) is the highest in the initial charging phase (Fig. 6b) and gradually decreases as the voltage on the capacitor increases. As can be seen, the initial charging current is limited by the series resistance of the capacitor R = 12 mΩ and I = 405 A. This is the current value that cannot be obtained from a photovoltaic cell. Therefore, a system for charging the supercapacitor with a constant current (e.g. I_Z_ = 250 mA) should be constructed independently of the resistance R. Figure 6c shows a regulated current source (using resistance R3) powered by voltage V_1_ = 10 V, supplying a constant current I_Z_ = 50 ÷ 500 mA to the load, which is a battery of series-connected supercapacitors (SC1, SC2) (Fig. 6d,e). The voltage balancing system (Fig. 6e) prevents the rated voltage V_RV_ of supercapacitors from being exceeded, which could result in the occurrence of electrolysis leading to their damage. This system is an active system based on Zener diodes (D2, D3), MMBZ5222B with a Zener voltage of 2.4 V which, along with resistors R9 and R10, form a system with non-linear characteristics (Fig. 6f—part b). Figure 4f shows that the I_DZ_ current flowing through the Zener diode D2 after exceeding the rated voltage of 2 × V_RV_ is approximately 6 mA. This allows current to flow freely between capacitors while equalizing voltage levels. This current is approximately 500 times greater than the leakage current I_nlc_ of the supercapacitor, which is typically of the order of 20 × 10^–6^ A.

**Figure 7:**
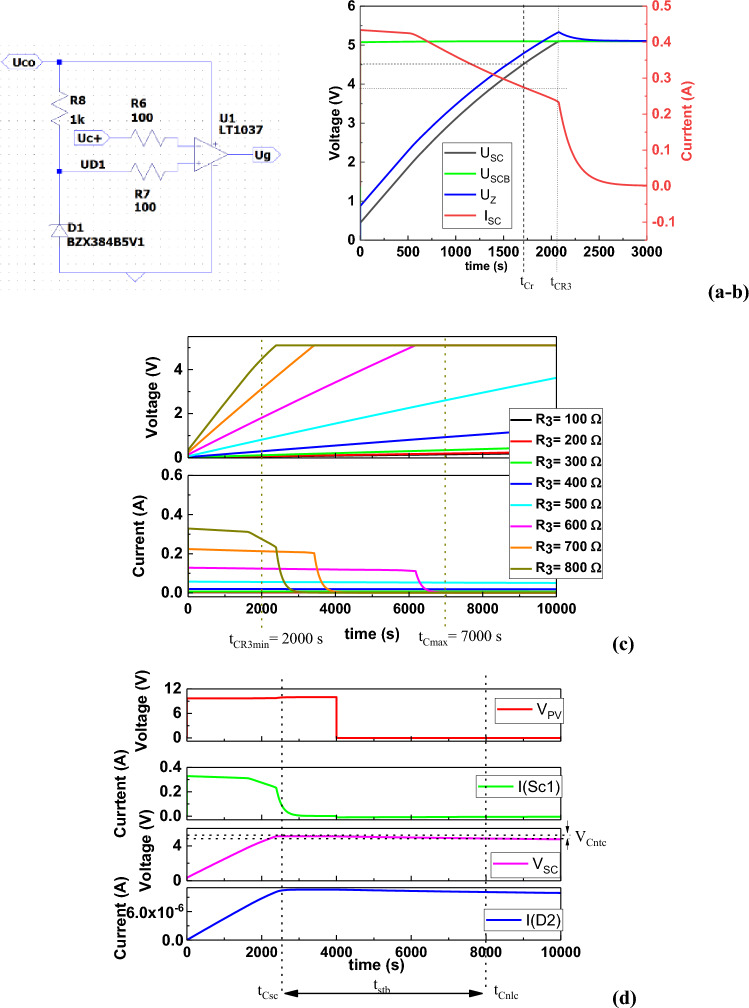
Supercapacitor charging voltage control system (**a**), characteristics of currents and voltages in the charging system of supercapacitor storage with constant current (**b**), current–voltage characteristics of the R3 function in the direct current supercapacitor charging system (**c**) and simulation of current and voltage characteristics in the direct current supercapacitor charging system (R3 = 900 Ω), where: V_PV_: photovoltaic battery voltage; I_SC_: supercapacitor battery charging current; V_SC_: charging voltage of supercapacitor storage, I_nlc_: supercapacitor leakage current; V_sctc_: voltage drop on the supercapacitor bank caused by the supercapacitor leakage current; t_Csc_: supercapacitor battery charging time; t_Cnlc_: time after which the stored energy in the supercapacitor bank will decrease by 10%.

Figure 7a shows the supercapacitor charging voltage control system. This system is designed to disconnect the supercapacitors from the charging source with the key (M1) after reaching the voltage V_SCB_ = 2 × V_RV_ = 4.8 V. The analysis of the supercapacitor charging system with constant current showed that such a system can successfully cooperate with a battery of photovoltaic cells that can provide a current I_nom_ of 1.2 A and a voltage V_nom_ = 10 V. Figure 7b shows the current and voltage characteristics of the supercapacitor battery charging system, and Fig. 7c shows the current–voltage characteristics of the R3 function.

Figure 7c shows that by changing the resistance value R3 from 400 to 1000 Ω the current can be adjusted from 20 to 500 mA, and thus the supercapacitor battery charging time from t_CR3min_ = 2000s. The time t_Cmax_ = 7000 s is the time of turning off the voltage source during the simulation and is equivalent to the time of exposure of photovoltaic modules to solar radiation. Figure 7d shows a simulation of the charging system taking into account the leakage current of I_nlc_ supercapacitors. The following values can be extracted from the chart: V_PV_ = 10 V, I_SC_ = 300 mA, V_SC_ = 5 V, I_nlc_ = 10 μA, t_Csc_ = 2450 s, t_Cnlc_ = 8000 s, V_sctc_ = 0.25 V. Moreover, the energy supplied (E_SC_) to the supercapacitor bank and the energy possible to be taken from the supercapacitor bank (E_SCZ_) when it decreases by 10% after t_Cnlc_ = 8000 s, and t_Cnlc_ = 2 h 20 min are equal to, respectively:

16 $$ {\text{E}}_{{{\text{SC}}}} = {\text{ V}}_{{{\text{sc}}}}^{{2}} \times {\text{ C}}/{2 } = {\text{ 1875 Ws}} $$

17 $$ {\text{E}}_{{{\text{SCZ}}}} = \, \left( {{\text{V}}_{{{\text{sc}}}} - {\text{ V}}_{{{\text{sctc}}}} } \right)^{{2}} \times {\text{ C}}/{2 } = { 1687}.{\text{5 Ws}} $$

While, the energy that can be taken from the supercapacitor bank when the voltage drops to V_SCp_ = 4.5 V, i.e. the limit voltage of the receiver's power supply is:

18 $$ {\text{E}}_{{{\text{SCp}}}} = \, \left( {{\text{V}}_{{{\text{sc}}}} - {\text{ V}}_{{{\text{sctc}}}} - {\text{ V}}_{{{\text{scz}}}} } \right)^{{2}} \times {\text{ C}}/{2 } = { 4}.{\text{7 Ws}} $$

Assuming that the receiver requires a supply voltage of V_omin_ = V_SCp_ = 4.5 V with a supply current of I_o_ = 1 A (e.g. SOS transmitter with a power of approx. 4 W), the time for which the signal will be sent can be calculated using the following formula:

19 $$ {\text{E}}_{{{\text{SCp}}}} = \, \left( {{\text{V}}_{{\text{o}}} \times {\text{ I}}_{{\text{o}}} } \right) \, \times {\text{ t}}_{{\text{p}}} $$

20 $$ {\text{t}}_{{\text{p}}} = {\text{ E}}_{{{\text{SCp}}}} / \, \left( {{\text{V}}_{{\text{o}}} \times {\text{ I}}_{{\text{o}}} } \right) \, = { 1}.0{\text{4 s}} $$

where: t_p_: effective operation time of the receiver of energy stored in the supercapacitor bank.

This means that an example SOS transmitter with operating voltage V_o_ = 4.5 V and current I_o_ = 1 A can send a signal in 1 s.

Since it was assumed that the operating voltage limit of the stored energy receiver V_SCp_ = 4.5 V and the voltage of the fully charged energy storage (supercapacitor bank) V_SC_ = 5 V, the time after which the charge will take place to V_SC_ = 5 V can be determined (see Fig. 7b).

21 $$ {\text{t}}_{{\text{d}}} = {\text{ t}}_{{{\text{CR3}}}} - {\text{t}}_{{{\text{Cr}}}} \to {\text{t}}_{{\text{d}}} = { 4}00{\text{ s}} \to {\text{t}}_{{\text{d}}} = {\text{ 6 min 4}}0{\text{ sec}} $$

Analyzing the entire charging cycle of the supercapacitor storage from time t_o_ = 0 to the time when they are fully charged t_csc_ = 2400 s (Fig. 7d) and then sending SOS signals, the considered SOS transmitter (energy receiver with supply voltage V_o_ = 4.5 V and supply current I_o_ = 1 A) could start its work after t_csc_ = 40 min 57 s from the time the photovoltaic battery was exposed to solar radiation and transmit SOS signals for t_p_ = 1 s every t_d_ = 6 min 40 s. However, exposure breaks resulting from self-discharge of supercapacitor storage caused by leakage current cannot be longer than t_stb_ (see Fig. 7d):

22 $$ {\text{t}}_{{{\text{stb}}}} = {\text{ t}}_{{{\text{Cnlc}}}} - {\text{ t}}_{{{\text{CSC}}}} \to {\text{t}}_{{{\text{stb}}}} = 1{\text{ h }}30 \, \min $$

**Figure 8:**
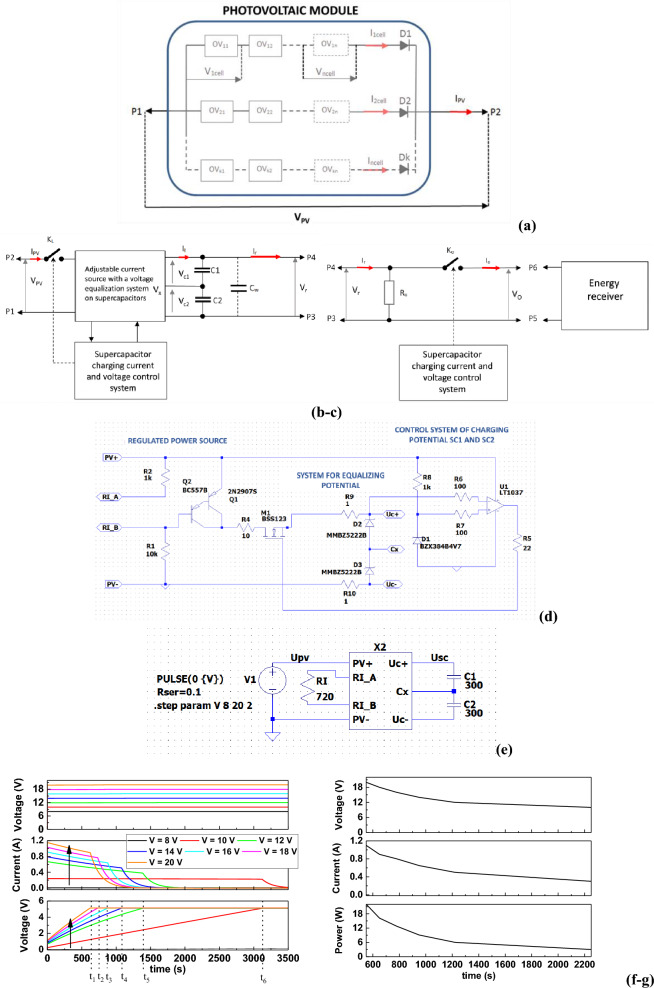

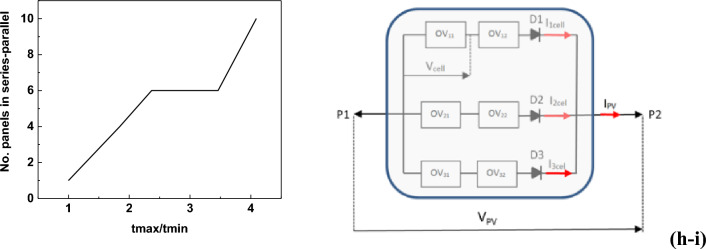
(**a**) Photovoltaic panel (battery of photovoltaic cells connected in series and parallel), (**b**) Supercapacitor charging system, C_w_: resultant capacity of the supercapacitor C_w_ = C/n; (**c**) supercapacitor discharge system, (**d**) schematic of the X2 model of the supercapacitor charging system in LTspice, (**e**) schematic of the supercapacitor charging system in LTspice using the X2 model. RI resistor setting the current of the current source, (**f**) charging characteristics of the supercapacitor in LTspice depending on the parameters of the PV panel, (**g**) supercapacitor charging time depending on the PV panel parameters, (**h**) characteristics of supercapacitor charging time optimization depending on the number of PV panels, and (**i**) optimal photovoltaic panel (modules of 20 photovoltaic cells connected in series and parallel in a 2 × 3 configuration), where: O11 ÷ O32—modules of 20 photovoltaic cells.

Figure 8a shows a schematic diagram of a photovoltaic panel in a series–parallel configuration. Single photovoltaic module with an output voltage V_cell_ and current I_cell_ in the following configuration form an active electric doubler, at whose terminals (P1, P2) the voltage and current can be received:

23 $$ {\text{V}}_{{{\text{PV}}}} = {\text{ V}}_{{{\text{1cell}}}} + {\text{ V}}_{{{\text{2cell}}}} + {\text{ V}}_{{{\text{3cell}}}} \cdots + {\text{ V}}_{{{\text{ncell}}}} ) \, - {\text{ V}}_{{\text{d}}} $$

24 $$ {\text{I}}_{{{\text{PV}}}} = {\text{ I}}_{{{\text{1cell}}}} + {\text{ I}}_{{{\text{2cell}}}} + {\text{ I}}_{{{\text{3cell}}}} \cdots + {\text{ I}}_{{{\text{ncell}}}} $$

where: V_d_ ≈ 0.6 V diode forward voltage with current I_cell_.

In such a system, the current flowing in the series branch has the same value. Diodes D1, D2, and D2 counteract the sudden drop in the PV panel current when any segment (serial branch) of the panel is darkened. According to the theory, the current in the darkened branch drops to the so-called dark current of a single cell. Figure 8b–c show block diagrams of the supercapacitor charging and discharging system, while Fig. 8d shows a diagram of the X2 model of the supercapacitor charging system (in LTspice), which was used to create the supercapacitor charging system (Fig. 8e). This system was used to determine the charging characteristics of the supercapacitor depending on the parameters of the PV panel (Fig. 8f,g).

From the graphs presented in Fig. 8f, it can be seen that when the V_PV_ voltage increases (a larger number of elements in the series branch) with a simultaneous increase in current efficiency (a larger number of series branches connected in parallel), the charging and recharging times of the supercapacitor shorten. Table 3 shows the numerical values of V_PV_ and I_PV_ read from the graph in Fig. 8f, depending on characteristics presented in Fig. 8g were plotted.

**Table 3:** The numerical values of V_PV_ and I_PV_ read from Fig. 8f.

Time	[s]	V_PV_	I_PV_	P_PV_
		[V]	[A]	[W]
t1	550	20	1.1	22
t2	650	18	0.9	16.2
t3	780	16	0.8	12.8
t4	950	14	0.65	9.1
t5	1220	12	0.5	6
t6	2250	10	0.3	3

From the graphs in Fig. 8g, it can be seen that the charging time of the supercapacitor is inversely proportional to the V_PV_ voltage and I_PV_ current of the PV panel. Characteristics of supercapacitor charging time optimization depending on the number of PV panels are shown in Fig. 8h and Table 4.

**Table 4:** Optimization of supercapacitor charging time depending on the number of PV panels.

t_max_/t_min_	Number of panels		
	Series	Parallel	Series–parallel
1.0	1	1	1
1.8	2	2	4
2.4	2	3	6
2.9	2	3	6
3.5	2	3	6
4.1	2	5	10

From Fig. 8i it can be seen that a significant change in the charging time of supercapacitors occurs when 6 photovoltaic modules are used. Each battery consists of 20 photovoltaic cells. The optimal number of 6 photovoltaic modules in a series–parallel configuration (2 × 3) makes up a PV panel. Our analyses have shown that the parameters of the optimal PV panel are: V_PV_ = 20 V; I_PV_ = 0.9 A; P_PV_ = 16.2 W. The charging time of the supercapacitor bank (2 capacitors connected in series) using the PV panel shown in Fig. 8i is t_łsc_ = 650 s (10 min 50 s), i.e. it has been shortened by 3.5 times compared to the basic photovoltaic battery consisting of 20 photovoltaics cells. The charging time for supercapacitor storage was also shortened presented in Table 5.

**Table 5:** The charging time for supercapacitor storage.

First charge time [s]		Top-up time from 4.5 to 4.8 V [s]
t1	550	40
t2	650	50
t3	780	60
t4	950	70
t5	1220	80
t6	2250	100

### Visualization of the physical model in graphical form using computer-aided design (MathCAD program)

**Figure 9:**
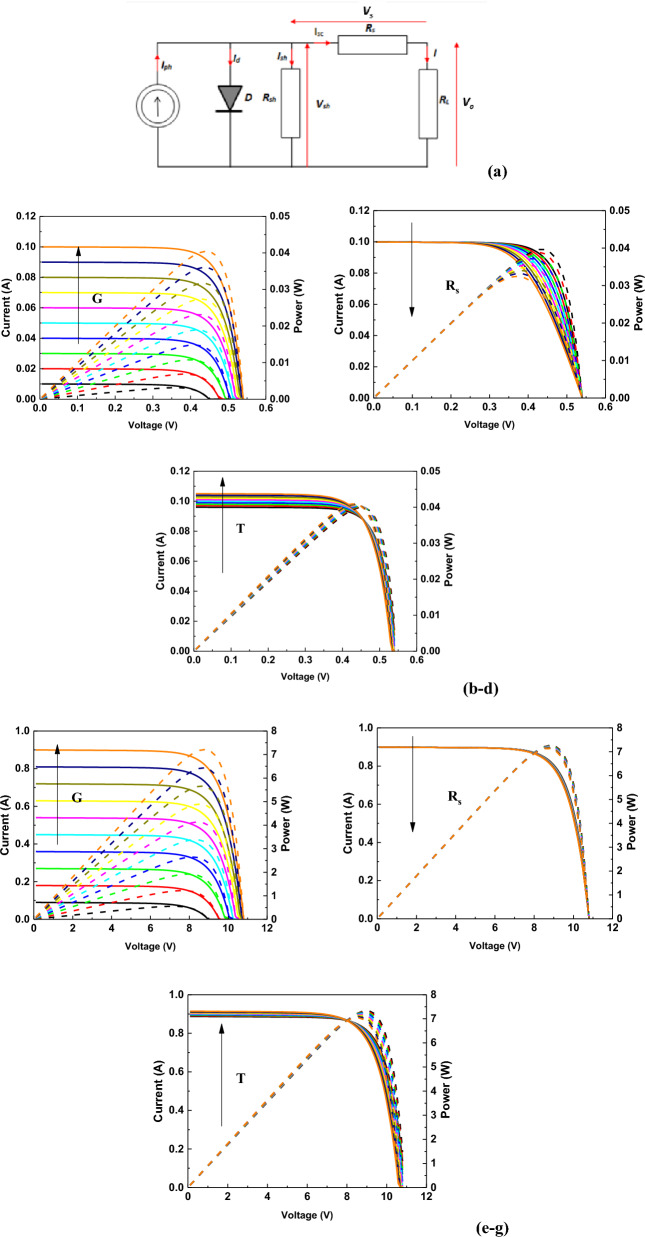
(**a**) The equivalent single-diode model of a photovoltaic cell, (**b**) characteristics of the influence of solar radiation, (**c**) series resistance, and (**d**) temperature, on the current, voltage and power values of single photovoltaic cell, solar panel characteristics under the influence of (**e**) sunlight intensity, (**f**) series resistance and (**g**) temperature.

The equivalent single-diode model of a photovoltaic cell with the influence of solar radiation (G), series resistance (R_s_), and temperature on the current, voltage, and power values is shown in Fig. 9.

A characteristic shift to the left can be observed in Fig. 9d which means that when the temperature increases (marked with an arrow), the output power decreases. The effects of other parameters (see the effect of changing R_s_ and I_ph_ in Fig. 9) are also consistent with the photovoltaic theory. Hence, it can be concluded that the above simulation can help to create a mathematical model of any PV panel based on photovoltaic cells operating in a series–parallel system.

Before visualizing the physical model in graphical form using computer-aided design, modeling of the solar characteristics of a battery of photovoltaic cells in a series–parallel system was carried out using a mathematical model. In Eq. 10, describing a photovoltaic cell (mathematical model), the configuration of photovoltaic cells was simulated by the flow of currents in the equivalent diagram of the PV panel (in accordance with Kirchhoff's first law) giving the Eq. 11.

The maximum solar radiation power density G_0_ of 1000 W/m^2^ was used to plot the characteristics presented in Fig. 9. While, the solar radiation power density was converted into units of illuminance, knowing that 1 W/m^2^ is equal to 0.0079 lx. An equivalent model of a PV module composed of single photovoltaic cells was developed based on the mathematical formulas and physical constants presented in the Materials and Methods section.

**Figure 10:**
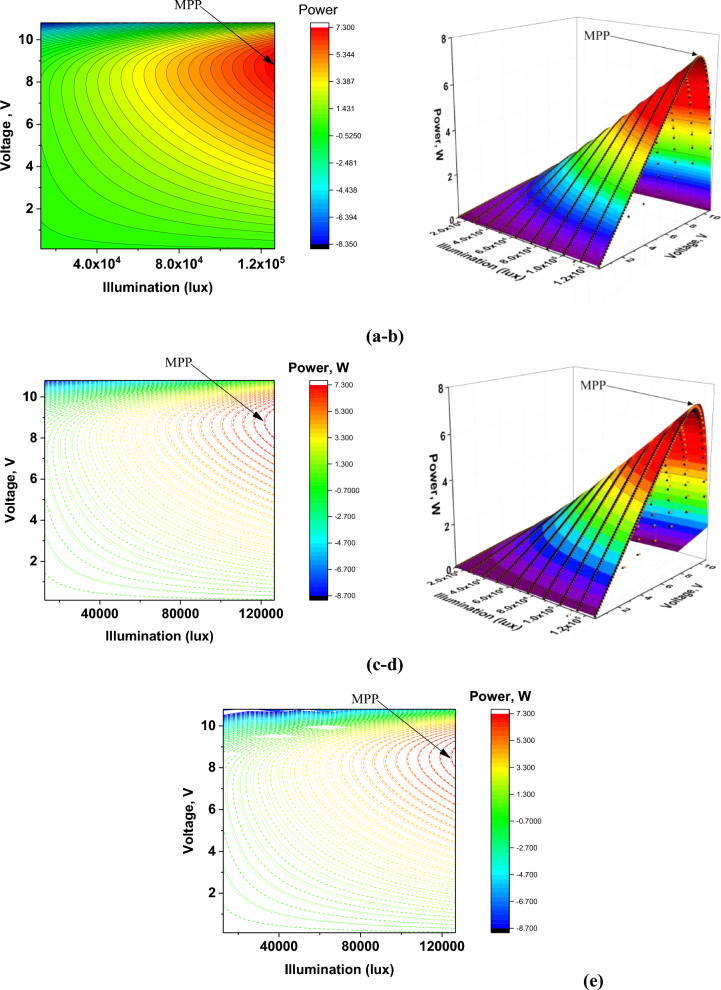
(**a**) Surface chart of PV panel power depending on illumination intensity with the optimal MPP, (**b**) 3D surface graph of PV panel power depending on illumination intensity with optimal MPP, (**c**) Surface chart of PV panel power depending on lighting intensity with the optimal MPP, (**d**) 3D surface chart of PV panel power depending on lighting intensity with the optimal MPP, (**e**) Surface chart of PV panel power depending on lighting intensity with the optimal MPP, where: V_p_ = 10.8 V, E_p_ = 1.266 × 10^5^ lx, V_div_ = 1.08 V, E_div_ = 1265.823 lx, R_s_: series resistance, T: temperature.

The next part of the research was the development of functional surface and 3D maps of PV panels working in a series–parallel system, also using the illumination intensity parameter. This presentation of individual dependencies, the influence of series resistance R_s,_ and temperature T on the optimal maximum power point (MPP) was intended to facilitate the analysis of the size of the phenomenon and the analyzed process. The obtained results are presented in Fig. 10.

**Figure 11:**
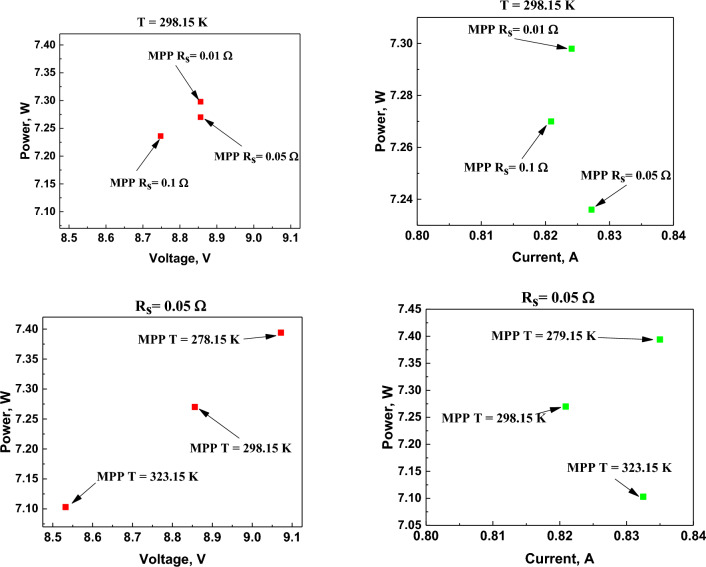
Graphical representation of MPP changes depending on the series resistance and operating temperature of the PV panel.

Table 6 illustrates the influence of the analyzed quantities, such as series resistance R_s_ and temperature T, on the maximum power point MPP, and Fig. 11 shows a graph of MPP changes depending on the series resistance and operating temperature of the PV panel.

**Table 6:** The influence of series resistance R_s_ and temperature T on the maximum power point MPP.

E_p_ = 1.266e + 005 lx				
Maximum power point	Parameter	MPP_opt_	V_opt_	I_opt_
		[W]	[V]	[A]
MPP	T = 298.15°K R_s_ = 0.05 Ω	7.27	8.856	0.8209
MPP_R_smin_	T = 298.15°K R_s_ = 0.01 Ω	7.298	8.856	0.8241
MPP_R_smax_	T = 298.15°K R_s_ = 0.1 Ω	7.236	8.748	0.8272
MPP_T_min_	T = 278.15°K R_s_ = 0.05 Ω	7.394	9.072	0.835
MPP_T_max_	T = 323.15°K R_s_ = 0.05 Ω	7.103	8.532	0.8325

### Characterization of solar charger based on silicon and dye-sensitized solar cells and supercapacitors

**Figure 12:**
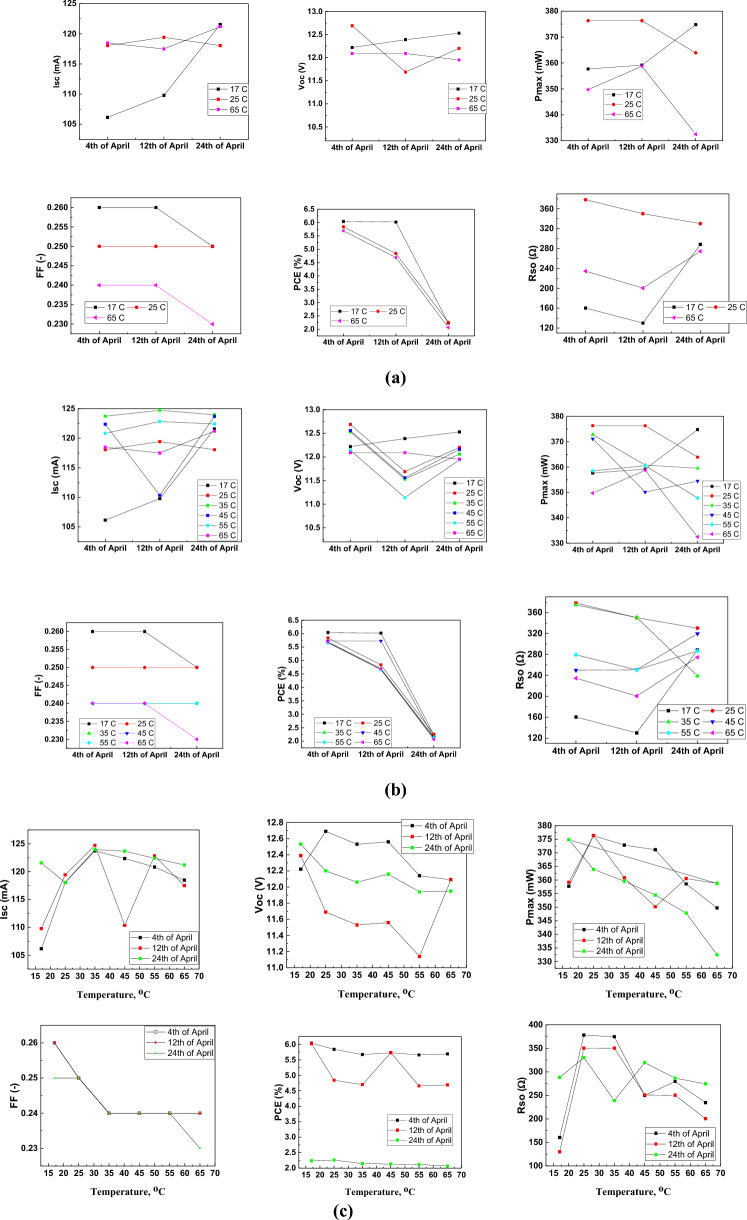
(**a**) The change in selected electrical parameters at temperatures of 17 °C, 25 °C, and 65 °C based on the three independent measurement sessions. (**b**) Change in selected parameters for all temperatures derived from three measurement sessions depending on the day of measurement. (**c**) The influence of temperature on selected parameters during measurements performed in the individual measurement sessions.

In the final stage, the selected silicon and dye-sensitized solar cells and supercapacitors were used to construct and investigate in-deep the solar chargers (see Experimental part). To measure the stability of electrical parameters over time and their temperature dependence, the constructed hybrid panel was tested for ten days. The electrical parameters and current–voltage characteristics were measured three times at the following temperatures: 17, 25, 35, 45, 55, and 65 °C. The measurement results are presented in Table 7. Figure 12a shows the change in selected electrical parameters for three temperatures: low (17 °C), RT (25 °C), and elevated (65 °C). The parameters were analyzed during three independent measurement sessions. Figure 12b shows the change in PV parameters for all temperatures and all measurement sessions depending on the day of measurement, whilst Fig. 12c shows the change in parameters depending on temperatures during measurements conducted in the individual measurement sessions.

**Table 7:** Electrical parameters of investigated PV modules at various temperatures and days in 2023.

Temperature	Parameter	04th April	12th April	24th April
17 °C	I_sc_ [mA]	106.15	109.79	121.56
	V_oc_ [V]	12.22	12.39	12.53
	I_max_ [mA]	86.11	89.01	94.12
	V_max_ [V]	4.09	4.04	3.98
	P_max_ [mW]	357.69	359.18	374.81
	FF [–]	0.26	0.26	0.25
	PCE [%]	6.04	6.02	2.24
	R_s_ [Ω]	160.34	130.13	288.24
	R_sh_ [Ω]	93.40	90.19	521.69
25 °C	I_sc_ [mA]	118.05	119.42	118.05
	V_oc_ [V]	12.69	11.69	12.20
	I_max_ [mA]	94.52	95.52	91.84
	V_max_ [V]	3.98	3.98	3.97
	P_max_ [mW]	376.33	376.33	363.93
	FF [–]	0.25	0.25	0.25
	PCE [%]	5.84	4.84	2.26
	R_s_ [Ω]	378.16	350.16	330.19
	R_sh_ [Ω]	250.65	255.65	76.74
35 °C	I_sc_ [mA]	123.72	124.72	123.96
	V_oc_ [V]	12.53	11.53	12.06
	I_max_ [mA]	46.11	45.11	98.09
	V_max_ [V]	8.09	9.09	3.67
	P_max_ [mW]	372.83	360.83	359.54
	FF [–]	0.24	0.24	0.24
	PCE [%]	5.67	4.70	2.15
	R_s_ [Ω]	374.53	350.53	238.97
	R_sh_ [Ω]	89.96	120.96	292.06
45 °C	I_sc_ [mA]	122.36	110.36	123.68
	V_oc_ [V]	12.56	11.56	12.16
	I_max_ [mA]	45.58	40.58	97.20
	V_max_ [V]	8.14	7.14	3.65
	P_max_ [mW]	371.15	350.15	354.47
	FF [–]	0.24	0.24	0.24
	PCE [%]	5.73	5.73	2.13
	R_s_ [Ω]	249.74	250.74	319.49
	R_sh_ [Ω]	393.78	380.78	188.82
55 °C	I_sc_ [mA]	120.83	122.83	122.40
	V_oc_ [V]	12.14	11.14	11.94
	I_max_ [mA]	44.17	40.17	97.13
	V_max_ [V]	8.14	7.91	3.58
	P_max_ [mW]	358.55	360.55	347.84
	FF [–]	0.24	0.24	0.24
	PCE [%]	5.66	4.66	2.12
	R_s_ [Ω]	279.37	250.37	286.56
	R_sh_ [Ω]	183.87	190.87	253.07
65 °C	I_sc_ [mA]	118.47	117.50	121.20
	V_oc_ [V]	12.09	12.09	11.95
	I_max_ [mA]	40.76	44.76	94.67
	V_max_ [V]	8.58	7.88	3.51
	P_max_ [mW]	349.75	358.75	332.50
	FF [–]	0.24	0.24	0.23
	PCE [%]	5.69	4.69	2.07
	R_s_ [Ω]	234.54	200.54	274.46
	R_sh_ [Ω]	263.31	310.31	309.99

Presented experimental results demonstrated that the hybrid solar chargers displayed some slight decrease in the overall performance over time at the lowest tested temperature. Taking into account, however, that the values of V_oc_ remain at similar levels, I_SC_ and P_max_ values showed improvement suggesting the stability of the hybrid charger over time. When it comes to the fill factor, which characterizes the shape of the I–V curve, it showed a slight decrease only in the case of T = 65 °C, thus suggesting a flattering tendency of the electric characteristics of the charger. This change affected also the power conversion efficiency (PCE) which in all cases displayed a significant decrease tendency. This behavior might be related to the presence of the DSSC modules, i.e. the presence of liquid electrolytes is the most vulnerable part. This would also explain the observed increase in series resistance of the photovoltaic module. Considering a general tendency of temperature influence, however, one can conclude that temperature did not have a significant impact on the charger performance and showed the same change in the overall performance over time.

**Figure 13:**
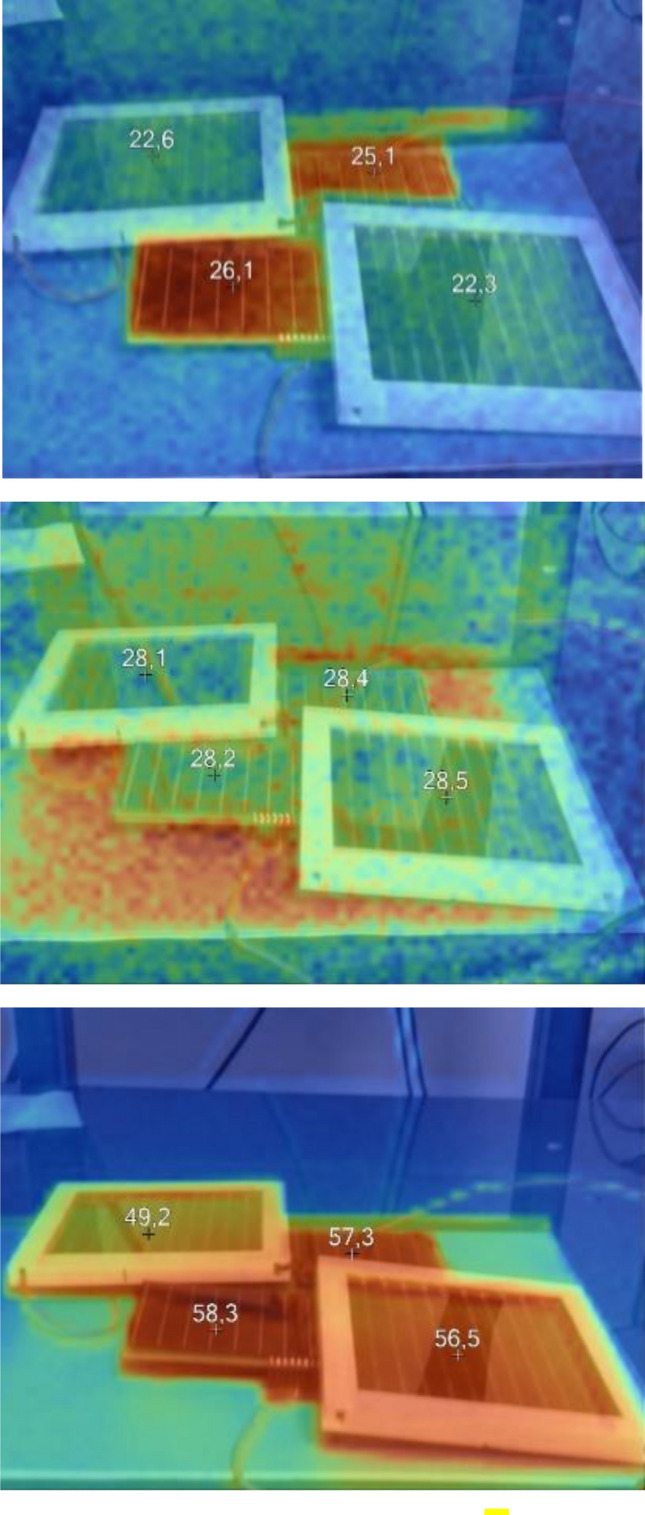
Thermograms of a photovoltaic DSSC-silicon panel at selected temperatures: 17 °C, 25 °C, and 65 °C, respectively.

Finally, temperature measurements for the constructed modular panels were performed using a thermal camera Fig. 13 shows an example of thermograms for photovoltaic panels at selected temperatures, whereas Table 8 shows the average parameters obtained for these temperatures during the individual measurement sessions.

**Table 8:** The average parameter values obtained at the selected temperatures during the individual measurement sessions of modular solar cells.

Date	T = 17 °C								
	I_sc_ (mA)	V_oc_ (V)	I_max_ (mA)	V_max_ (V)	P_max_ (W)	FF [–]	PCE (%)	R_s_ (Ω)	R_sh_ (Ω)
2023-04-04	106.15	12.22	86.11	4.09	357.69	0.26	6.04	160.34	93.4
2023-04-12	109.81	12.392	89.0075	4.0375	359.1755	0.26	6.015	130.1285	90.192
2023-04-24	121.56	12.53	94.12	3.98	374.81	0.25	2.24	288.24	521.69
T = 25 °C									
2023-04-04	118.05	12.2	91.84	3.97	363.93	0.25	2.26	330.19	76.74
2023-04-12	119.42	11.69	95.52	3.98	376.33	0.25	5.84	376.16	255.65
2023-04-24	118.05	12.2	91.84	3.97	363.93	0.25	2.26	330.19	76.74
T = 65 °C									
2023-04-04	117.5	12.09	40.76	7.88	349.75	0.24	5.69	234.54	263.31
2023-04-12	117.47	12.09	44.76	8.58	358.75	0.24	4.69	200.54	310.31
2023-04-24	121.2	11.95	94.67	3.51	332.5	0.23	2.07	274.46	309.99

Taking into account the thermal images of the photovoltaic modules it is evident that the silicon-based modules showed a higher temperature by 4 °C at the temperature of 17 °C after exposure to the illumination source. At 25 °C and 65 °C, the temperature on the panels was very similar. The difference observed for one of the DSSC panels is related to the reflection of the daylight from the glass surface of the module which decreases the displayed value of the temperature.

Finally, preliminary assumptions (requirements) were developed concerning the electrical and optical parameters for new DSSC cells that could be used in future in the innovative solar charger. To sum up, DSSC cells that could operate with a solar charger based on supercapacitors should be characterized by: power density greater than E > 4 mW/cm^2^; short-circuit current of at least I_sc_ = 50 mA; open circuit voltage greater than V_oc_ > 6 V; maximum operating voltage of at least V_max_ = 4 V, and maximum operating current, at least I_max_ = 45 mA. In addition, DSSC should show absorbance in the range of 350–800 nm and the electrolyte filling the dye cell cannot show absorption in the DSSC operating range. After fulfilling the aforementioned parameters, DSSC can be a fully integral part of a solar charger based on supercapacitors that would meet the dimensional requirements of a photosensitive field smaller than 120 mm × 120 mm while maintaining the dimensions of the device, i.e. 150 mm × 150 mm × 60 mm. Of course, as in a charger made only on silicon cells, a single DSSC module should have a maximum size of the photosensitive field not larger than 50 mm × 50 mm. Then, the entire photovoltaic module would be built of four such cells operating in a series–parallel connection system. Such a module would be characterized by the following parameters: short-circuit current, at least I_sc_ = 100 mA; open circuit voltage greater than V_oc_ > 12 V; maximum operating voltage, at least V_max_ = 8 V; and maximum operating current, at least I_max_ = 90 mA. Such a module built only of DSSC would be a fully interchangeable element of the module built of silicon cells used in the charger as presented in our previous work ^15^. A solar charger constructed from new dye solar cells could successfully serve as a handy portable power supply device providing access to electricity for small devices, e.g. tablets, smartphones, GPS receivers, handheld communication systems, or SOS transmitters in emergencies and field applications.

## Conclusions and future

We have presented a new approach for the construction of a modular solar charger based on both silicon solar cells, dye-sensitized solar cells (DSSC), and supercapacitors. The validity of the concept was successfully verified via applied experimental and theoretical (the MathCAD program) diagnostic tools. It was observed that despite the difference in the electric characteristics of both solar panels under study, it was possible to efficiently combine both technologies and to benefit from their individual advantages.

Our research can be summarized as follows:

- The supercapacitor bank charging system (2 supercapacitors with a capacity of 300 F each in series) forms a regulated current source.
- For a receiver requiring a supply power approx. 4 W, the optimal photovoltaic module should have the following parameters: voltage V_pv_ = 20 V; current I_pv_ = 0.9 A; and power P_pv_ = 16.2 W,
- For given configurartion the energy supplied to the supercapacitor bank in the first charge requires E_SC_ = 1875 W,
- The energy that can be extracted from the supercapacitor bank, including decreases of 10% (impact of self-discharge), was found E_SCZ_ = 1687.5 W,
- The time of first charging of supercapacitor storage was equal t_csc_ = 40 min 57 s,
- The effective operation time of the receiver of energy stored in the supercapacitor bank was t_p_ = 1.04 s,
- The time after which the V_SC_ is recharged 5 V was t_d_ = 6 min 40 s,
- The exposure breaks resulting from self-discharge of supercapacitor storage caused by leakage current cannot be longer than t_stb_ = 1 h 30 min,
- The silicon-based modules showed a higher temperature by 4 °C at 17 °C, after exposure to the illumination source, and at 25 °C and 65 °C for the surrounding temperature was similar,
- Comparative analysis of solar chargers based on different photovoltaic cells showed an increase in electrical parameters for the proposed modular inorganic–organic cells compared to DSSC on a rigid substrate.

Additionally, it was evident that the DSSC gave resistance to the elevated temperature when compared to only silicone panels. It is also clear that the DSSC technology based on the liquid electrolyte is not the final solution, however, overcoming this issue would drastically change its applicability in the future.

## Data Availability

The authors declare that the data supporting the findings of this study are available within the paper.

## List of symbols

AI: Artificial intelligence
C_w_: Resultant capacity of the supercapacitor battery
D: Diode
DSSC: Dye-sensitized solar cell
E: Energy
E_SC_: Energy supplied to the supercapacitor bank
E_SCZ_: The energy possible to be taken from the supercapacitor bank
FF: Fill Factor
G_0_: The maximum solar radiation power density
E_0_: The maximum of light intensity
I: Current of the PV panel
I_d_: Diode current
I_max_: Maximum current
I_o_: Diode saturation current
I_ph_: Current generated by the photovoltaic effect
I_nlc_: Supercapacitor leakage current
I_sc_: Short-circuit current and supercapacitor battery charging current
I_sh_: Current of the shunt resistance of the PV cell
ML: Machine learning
MPP: Maximum power point
n: Number of supercapacitors
PCE: Power conversion efficiency
P_max_: Maximum power
P_nom_: Nominal power point
PV: Photovoltaic
V_d_: Diode potential
V_max_: Maximum voltage
V_o_: Maximum charging voltage
V_oc_: Open circuit voltage
V_PV_: Photovoltaic battery voltage
V_SC_: Charging voltage of supercapacitor storage
V_sctc_: Voltage drop on the supercapacitor bank caused by the supercapacitor leakage current
V_sh_: Potential of the shunt resistance of the PV cell
R: Internal resistance of the capacitor
R_s_: Series resistance
R_sh_, R_p_: Shunt (parallel) resistance
T: Temperature
t_c_: Time of charging the supercapacitor
t_Cnlc_: Time after which the stored energy in the supercapacitor bank will decrease by 10%
t_Csc_: Supercapacitor battery charging time
t_p_: Effective operation time of the receiver of energy stored in the supercapacitor bank
τ: Capacitor charging constant
